# Computed tomography chest findings in post-acute COVID-19 lung disease at a South African regional hospital - a descriptive study

**DOI:** 10.11604/pamj.2023.44.175.39711

**Published:** 2023-04-13

**Authors:** Pieter Barend Kotzé, Ryan Manthey, Stephanie Griffith-Richards, Christelle Ackermann, Karl Klusmann

**Affiliations:** 1 Radiology Tygerberg Hospital, University of Stellenbosch, Stellenbosch, South Africa,; 2 Internal Medicine Department, Worcester Hospital, Worcester, South Africa,; 3 Department of Medicine, Stellenbosch University, Worcester Hospital, Worcester, South Africa

**Keywords:** Post-acute COVID-19 syndrome, COVID-19 pneumonia, pulmonary embolism, HIV, pulmonary fibrosis, computed tomography, tuberculosis

## Abstract

**Introduction:**

whilst many studies have focused on acute and chronic complications of COVID-19, few studies have been performed on the immediate post-acute COVID-19 phase complications. The objective of the study was to describe computed tomography (CT) imaging findings in patients from a South African (SA) cohort during the post-acute COVID-19 phase. To describe the findings using existing CT description systems and, if present, pulmonary imaging findings unique to our cohort.

**Methods:**

a review of CT chest examinations performed over the second wave of COVID-19 in SA for post-acute COVID-19 cardiorespiratory complaints at Worcester Hospital. The CT findings were described using a modified semi-quantitative tabulation method.

**Results:**

eight males and 12 females met the inclusion criteria with a mean age of 56 years. Half had hypertension, 11 had diabetes, two had human immunodeficiency virus (HIV), half had raised D-dimers and six had pre-existing lung disease. The predominant parenchymal pattern was mixed ground glass and reticular changes in a diffuse/peripheral multilobar distribution with relative sparing of the left upper lobe. Four cases demonstrated pulmonary emboli, 50% pulmonary hypertension, three pleural effusions and nine lymphadenopathy. None of the cases had evidence of active pulmonary tuberculosis.

**Conclusion:**

CT lung findings appear to mirror global findings with expected evolutionary differences. An interesting observation was the relative sparing of the left upper lobe. Reporting using the modified table proved efficient. Real-world extrapolation of our findings is limited by low-case numbers.

## Introduction

The first case of coronavirus disease 2019 (COVID-19) was reported on 1^st^ December 2019 in Wuhan, China [1]. To date (24^th^ November 2022), approximately 635,709,158 cases and 6,603,803 deaths have been reported (WHO). Whilst the advent of vaccinations and progressively less virulent strains have decreased the global morbidity and mortality of COVID-19, the initial waves had a profound worldwide impact [2].

As of November 24^th^, 2022, the National Institute for Communicable Diseases has reported that over 37.93 million vaccinations have been administered in South Africa, resulting in a significant decrease in both hospitalizations and mortality rates, particularly with newer variants and strains [3]. SARS-CoV-2 clinical manifestations share some similarities to other coronaviruses such as Middle East Respiratory Syndrome (MERS) and Severe Acute Respiratory Syndrome (SARS), the unique and evolving viral genome has led to unique clinical presentations during this pandemic. Diverse clinical presentations have been observed between the different waves [4].

While COVID-19 typically manifests in various organ systems, most hospitalisations have been due to respiratory complications, which include acute respiratory distress syndrome, venous thrombotic embolic disease and COVID-19 pneumonia [5]. COVID-19-related respiratory illness demonstrates characteristic radiological features, and increased severity is associated with prolonged hospitalisation and decreased survival [6,7]. Patients with cardiorespiratory complaints frequently undergo imaging investigations including plain radiography, ultrasound and chest computed tomography (CT). Acute clinical presentation and CT chest findings have predictive value for long-term outcomes [8,9]. A large proportion (85%) of patients hospitalised with moderate to severe COVID-19 pneumonia experience protracted symptoms, some even after discharge [10].

After day 28 of COVID-19, patients with persistent symptoms or complications enter the post-acute phase and may be diagnosed with post-acute COVID-19 syndrome [11]. Post-acute COVID-19 syndrome is defined as a multisystem syndrome characterized by persistent symptoms beyond four weeks from the onset of acute symptoms. Investigators have postulated a range of possible mechanisms including residual systemic inflammation, end-organ damage, prolonged ventilation, and secondary impact on pre-existing health conditions [12]. Although post-acute COVID-19 syndrome can affect every organ system, cardiorespiratory symptoms are common, with chest pain and dyspnoea present in 21% and 43% of patients respectively, prompting further investigations including imaging, serology, electrocardiography and electromyography [12,13].

Adult contrasted CT chest or CT pulmonary angiography in COVID-19 lung disease is typically performed in the following settings: hospitalised patients with acute cardiorespiratory deterioration and suspected pulmonary embolism [14]; patients with delayed cardiorespiratory recovery [8,15]; patients with initial improvement and discharge, who have recurrent symptoms and possible organising COVID pneumonia [16] and high-resolution computed tomography (HRCT) are performed when post-infective fibrotic lung disease is suspected. Many standardised reporting lexicons and grading systems have been developed for reporting on CT chest findings in COVID-19 pneumonia.

The rise in post-acute COVID-19 cases has prompted descriptive studies of the HRCT chest findings in this setting [17]. Correlation between these findings and the initial, as well as subsequent clinical presentations have been demonstrated [12]. Descriptive radiological studies have also demonstrated features both specific to COVID-19 respiratory illness and common to other viral lung diseases. Of note, similarities between the HRCT findings in post-viral pneumonia due to SARS and MERS have been documented [18,19]. The progressive features have similarities with those of progressive interstitial lung diseases (ILD) and organising cases of pneumonia [20]. Therefore the use of established CT descriptors for ILD may be appropriate, in particular when describing parenchymal changes [21]. Chest CT in these patients could aid in the diagnosis, prognostication and therapeutic interventions, as for other post-viral cases of pneumonia.

In resource-limited countries such as South Africa the patient load on the already strained healthcare system presents unique challenges, especially during the initial more severe waves of COVID-19. Increased demands on bed space, special investigations and treatment/intervention mean that these resources had to be allocated in unique and dynamic ways [22,23]. South Africa remains the global epicentre for HIV and TB. The impact of the COVID-19 pandemic on the background high prevalence of HIV and pulmonary tuberculosis (PTB) in South Africa has been a concern since the early stages of the pandemic. Studies are ongoing to determine the immediate and downstream effects of the COVID-19 pandemic on this unique population group [24].

This study aims to retrospectively describe CT chest examinations in patients scanned for cardiorespiratory complaints in the post-acute phase of their COVID-19 illness in a South African regional hospital. Resource limitations, in particular CT scan access and bed pressure, lower socio-economic background and endemic disease milieu may reveal unique disease patterns/complications. The objectives of the study are: to describe CT imaging findings in patients from a South African cohort during the post-acute COVID-19 phase, using existing CT description systems and, if present, pulmonary imaging findings unique to our cohort.

## Methods

**Study design and participants:** this was a retrospective, descriptive, cross-sectional study conducted at Worcester Regional Hospital (WRH), a 227-bed secondary-level hospital in the Cape Winelands a district of the Western Cape Province of South Africa. All adult patients (>18 years) who underwent CT chest examinations for post-acute COVID-19 cardiopulmonary-related indications during the period of 1^st^ November 2020 to 31^st^ March 2021 were included, thus sampling over the second wave in South Africa. Scans deemed technically inadequate were excluded from the study.

**Data collection:** customised searches of the WRH picture archive and communication System and radiology information system were conducted in conjunction with detailed analyses of the WRH medical records. All COVID-19 positive cases who underwent CT chest examinations during the post-acute COVID-19 phase for cardiorespiratory indications were identified and included.

Two consultant radiologists independently reported each CT chest in accordance with the International Fleischner Society guidelines. Any discrepancies in initial interpretation were resolved by consensus. Findings were captured utilizing a semi-quantitative tabulation method. CT chest findings were captured using a semi-quantitative tabulation method in which each lung lobe (with the lingula included with the left upper lobe) in terms of the predominant parenchymal change pattern, what percentage volume is involved, and whether the distribution is central, peripheral or diffuse was recorded. This method of capturing has been proven effective in describing the parenchymal disease in interstitial lung disease [20].

Other pertinent findings captured include the presence of pulmonary emboli, pleural disease, evidence of pulmonary hypertension/cor-pulmonale, pre-existing lung disease and pathognomonic features of PTB. Radiological criteria for pulmonary hypertension include pulmonary trunk enlargement (>30mm on axial imaging) and inverted pulmonary trunk to aortic ratio (ascending aorta < pulmonary trunk diameters). Pulmonary emboli demonstrated as pulmonary arterial filling defects on contrasted CT sequences. CT scans with features suggestive of underlying pre-existing lung disease were identified. To validate these findings, the principal investigator provided previous anonymized imaging and clinical data to the readers. Clinical and serological data captured for each case include age, HIV status, diabetes, hypertension, D-dimer levels, scan date and COVID-19 diagnosis date.

**Statistical analysis:** summary statistics were reported as frequencies and percentages.

**Data availability:** data pertaining to this research are available from the corresponding author.

**Disclaimer:** views and opinions expressed in this article are those of the authors and do not necessarily reflect the official policy or position of any affiliated agency of the authors.

**Ethical considerations:** ethical approval was obtained from the Health Research Ethics Committee of the University of Stellenbosch. (S21/10/016_COVID-19) and site approval was obtained from the hospital. Informed consent was not required for this retrospective, descriptive study.

## Results

**Demographics:** twenty-three cases met the initial inclusion criteria. Two CT studies were technically inadequate and one case was a folder duplicate error, thus leaving 20 cases eligible for interpretation. The included cases consisted of 8 males and 12 females with a mean age of 56 years. According to the NHLS database and clinical documents, HIV infection was present in 10% of the cases, absent in 75% and unknown in 15%. Ten patients were known with hypertension and 11 with diabetes. D-dimer tests were elevated in 50% of the cases (Table 1).

**Table 1:** demographics and clinical characteristics of sample

Variable	
**Age, mean (SD)**	56 (15.1)
Range	26 - 80
**Days of COVID (28+)**	36.7 (8.5)
Range	28 - 51
**Gender (female)**	12 (60%)
**Hypertension (yes)**	10 (50%)
**Diabetes (yes)**	11 (55%)
**HIV status**	
Negative	15 (75%)
Positive	2 (10%)
Unknown	3 (15%)
**Pre-existing lung disease (yes)**	6 (30%)
Bronchiectasis (including post-PTB)	3
COPD/Emphysema	2
Interstitial lung disease	1
**Predominant distribution**	
Central	1 (5%)
Diffuse	7 (35%)
Peripheral	12 (60%)
**Presence of pleural pathology (yes)**	3 (15%)
**Lymphadenopathy present (yes)**	9 (45%)
**Radiographically evident pre-existing disease present**	
Malignancy	0
Bronchiectasis	3 (15%)
COPD/Emphysema	3 (15%)
Interstitial lung disease	2 (10%)
**Markers of pulmonary thromboembolism**	
D-dimer (raised)	10 (50%)
CT evidence of PE	4 (20%)
CT evidence of pulmonary hypertension	10 (50%)

The scans were performed between 29 and 49 days after diagnosis of COVID-19 (day 0). These dates roughly correspond with patients who had prolonged complicated admissions and failed/complicated outpatient management. These scan dates are relatively early compared to other studies done in the post-acute phase.

**Figure 1:**
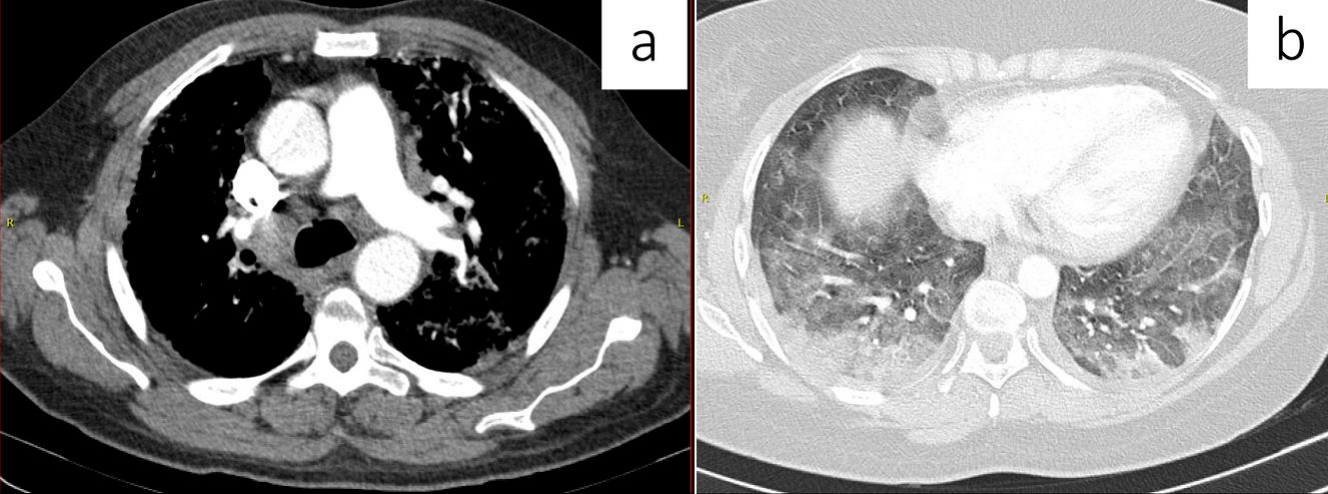
a) axial CT soft tissue window demonstrating mediastinal lymphadenopathy; b) axial CT lung window demonstrating bilateral pleural effusions with mixed ground glass and reticular changes

**Figure 2:**
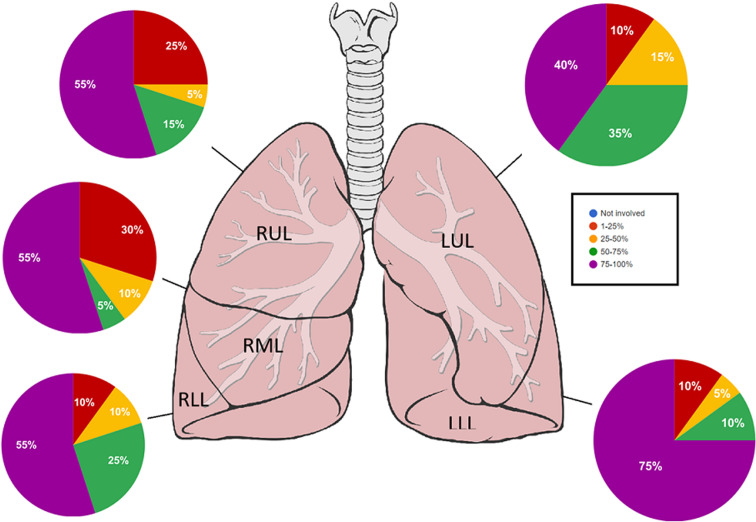
the amount of involvement of the parenchyma in terms of volume (expressed as a percentage of the lobe volume)

**Figure 3:**
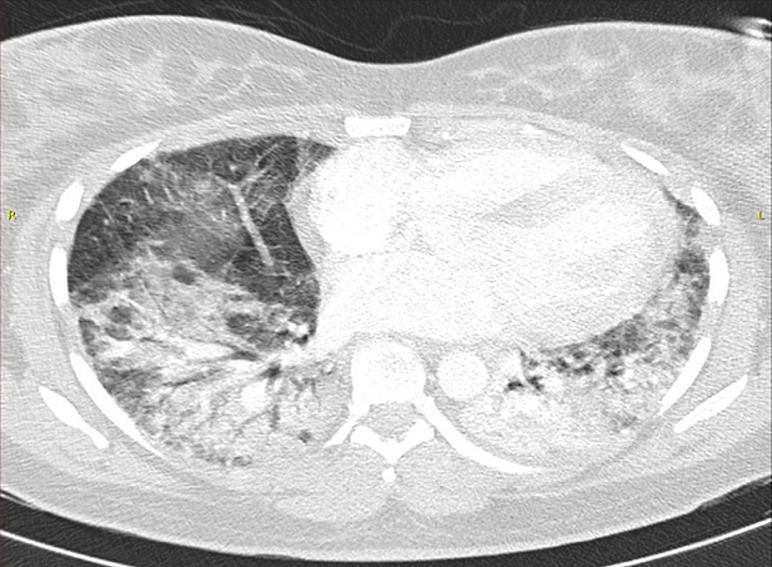
axial lung window CT demonstrating bilateral consolidation in bilateral posterior basal lower lobes

**Figure 4:**
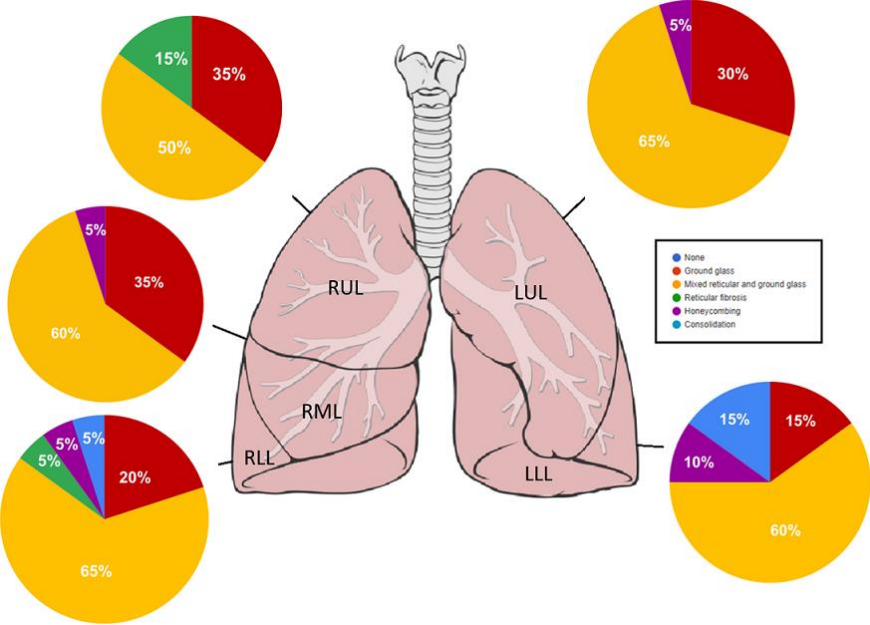
predominant pattern of parenchymal involvement

**Figure 5:**
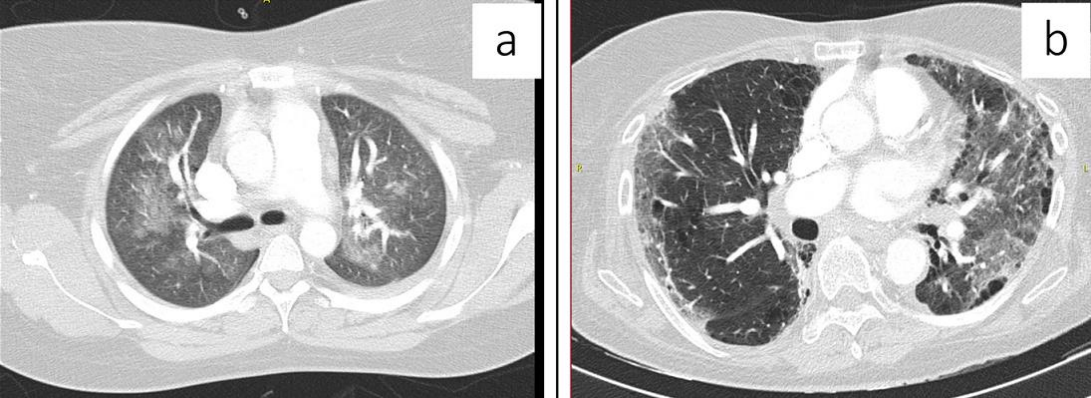
a) CT axial lung window demonstrating central distribution of ground glass changes; b) axial lung window CT demonstrating predominantly peripheral mixed reticulation and ground glass opacification

**Radiological findings:** thirty percent of the case reviewed demonstrated evidence of pre-existing lung disease. Pre-existing conditions included post-PTB lung disease, COPD, emphysema and interstitial lung disease not otherwise specified (NOS). None of the cases demonstrated CT evidence of active PTB. Nine cases demonstrated lymphadenopathy and 3 cases of pleural disease (effusions) (Figure 1). Only 20% of the cases demonstrated pulmonary emboli and 50% evidence of pulmonary hypertension. Most (75%) of the pulmonary emboli were central. Parenchymal changes were observed in all lobes in all of the cases with varying volumetric (percentage of lobe volume) involvement (Figure 2): right upper, middle and lower lobes predominantly demonstrated 75%-100% involvement; the left upper lobe demonstrated less extensive involvement with only 8 cases having more than 75% and 7 cases 50-75% volume involvement and the most extensively affected was the left lower lobe with more than 75% volume involvement in 15 (75%) of the cases. The predominant (50-65%) parenchymal pattern of involvement was mixed ground glass and reticular changes followed by ground glass opacification (GGO) (15-35%) (Figure 3). Consolidation, honeycombing and fibrosis were rarely seen as the predominant parenchymal pattern (Figure 4). The distribution of lung changes was predominantly peripheral (35%) and diffuse (60%) with only one case (5%) demonstrating a central distribution (Figure 5).

## Discussion

The typical CT chest findings in our post-acute COVID-19 cohort are characterised by peripheral bilateral predominantly mixed ground glass and reticular changes and/or pure GGO favouring the lower and middle lobes with relatively less extensive left upper lobe changes. Additional findings are CT evidence of pulmonary hypertension and lymphadenopathy. Rarely, pleural effusions are observed.

The semi-quantitative tabulation method used to capture the imaging findings proved to be effective in particular when describing parenchymal volume and predominant pattern involvement. The radiologist consultants performing the reads had few (three cases with minor changes with regard to the presence of pre-existing lung disease) discrepancies to address during the consensus reading.

Early post-acute COVID-19 parenchymal changes in our study appear to correlate with what is globally observed in post-acute phase studies. Compared to other studies, the mixed ground glass and reticular change are what can be expected following up on acute lung changes. In another study, ground glass opacification has been found in up to 75% of CT scans done at three months follow-up [25]. This is interesting as GGO is typically seen as a marker of acute inflammation in other viral cases of pneumonia.

Two main acknowledged pathogenic pathways are identified in severe COVID-19: Diffuse alveolar damage caused by direct viral toxic effect, potentially exacerbated by ventilator-induced lung injury, and an autoimmune/inflammatory mechanism leading to microvascular insults and organizing pneumonia-like changes [19]. Extensive initial ground glass changes have been found predictive of long-term follow-up fibrotic-like change and eventual true fibrosis. These long-term changes are deemed evolutionary and therefore in similar lung volume distribution seen in the acute phase [26].

**Figure 6:**
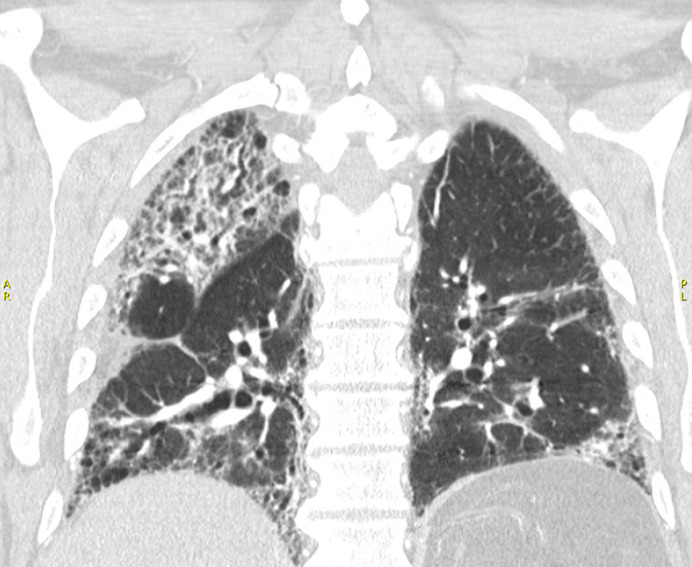
coronal CT lung window demonstrating mixed reticulation, fibrosis and honey combing; note the relative sparing of the left upper lobe

Our findings are therefore in keeping with a mixed acute lung injury pattern and evolutionary changes seen in other long-term follow-up studies. The relatively less extensive involvement of the left upper lobe correlates to what is was seen in one local study done in an acute phase COVID-19 cohort [27]. Proposed explanations for the relative left upper lobe sparing include physiological and anatomical regional asymmetries. Right upper lobe lymphatics drain to the right hilum and the left upper lobe to the left subclavian vein. According to scintigraphic perfusion studies, the right lung receives preferential perfusion relative to the left lung [28]. COVID-19 lung injury is partially caused by humoral and cell-mediated inflammation which in turn is dependent on systemic circulation. These factors, in the context of cardiac dysfunction in the critically ill, may lead to radiographic sparing of the left upper lobe in the acute phase of COVID-19 pneumonia (Figure 6).

**Figure 7:**
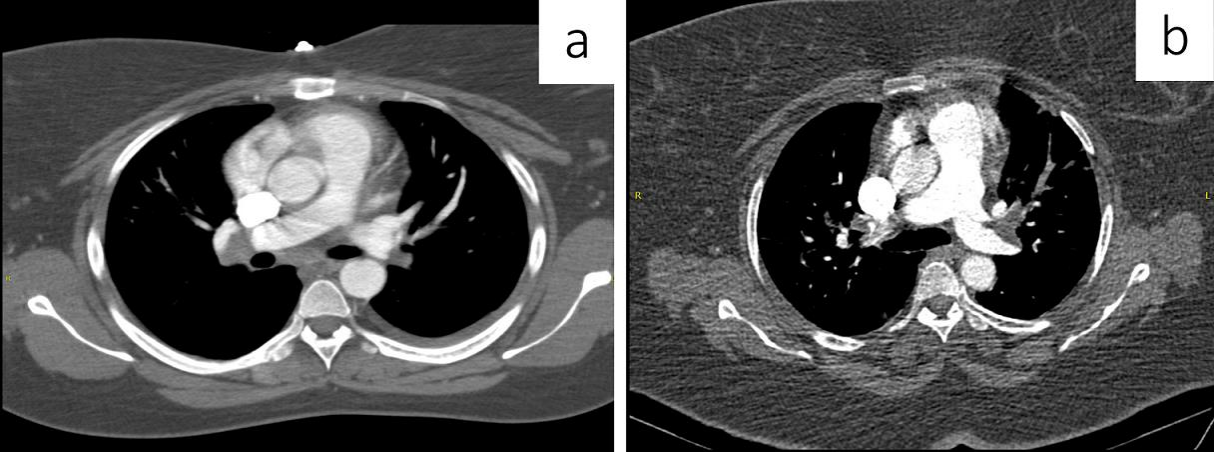
a) axial CT soft tissue window demonstrating bilateral central pulmonary emboli; b) axial CT soft tissue window demonstrating bilateral central pulmonary emboli

The prevalence of pulmonary emboli was lower in our cohort compared to local and international studies. A possible explanation for this could be the different timing and indications for the CT scans. In our study, the indications were often done in the context of a patient not recovering in the hospital and needing their first CT scan as part of their delayed recovery workup. These cases may be less likely to demonstrate pulmonary emboli as they would have been placed on aggressive thromboprophylaxis as part of their in-hospital treatment. International post-acute phase studies include large numbers of follow-up patients at a much later stage. It is unlikely that these patients were placed on aggressive prolonged outpatient thromboprophylaxis. Additionally, these patients likely suffered from debilitating post-acute COVID-19 symptoms leading to sedentary lifestyle adjustment. These factors may have predisposed their cohorts to an increased risk for pulmonary embolism (Figure 7).

The low prevalence of pre-existing disease may be explained by lower baseline functional status and strict admission criteria for high and intensive care units. Patients with poor baselines that survive acute COVID-19 are more likely to be discharged at lower functional statuses and are less likely to meet admission criteria to high and intensive care facilities or long admission units for rehabilitation [29].

The low prevalence of HIV-infected cases is unexpected given the high endemic prevalence and HIV being known to be an independent risk factor for severe COVID-19 [24]. It is likely that metabolic risk factors outweigh HIV as an independent risk factor and is more common. It may also be attributed to the statistically insignificant size of the cohort.

The incidence of hypertension and diabetes in our study population mirrored what is seen globally in cohorts admitted with severe COVID-19. None of the cases demonstrated active evidence of PTB. Patients who present with respiratory complaints to the South African health care system are typically screened for PTB early as part of their workup. Patients diagnosed with PTB may potentially not qualify for extended admission to high-care unit environments. Persistent cardiorespiratory symptoms may be attributed to PTB itself. Cases with PTB are therefore less likely to have been included in our study.

**Study limitations:** a significant limitation of this study was the low number of eligible cases included. Despite a high patient load during the third wave in South Africa, the number of scans performed on patients in their post-acute COVID-19 phase was low. The small sample size reduces the external validity of our findings to a larger South African population. This may be due to strict inclusion criteria, however global studies with similar criteria delivered much higher yields. The availability of CT scanners per capita is relatively high in first-world countries in which these studies were performed [23]. Clinician-radiologist combined threshold to perform CT scans as part of diagnostic workup will likely be influenced on the local availability of the resource. Diagnostic and treatment protocols are often shaped around local resource availability [30].

## Conclusion

The use of existing CT descriptive terms in the form of a semi-quantitative table with parenchymal pattern changes based on interstitial lung disease patterns proved effective during the data collection phase of the study. Very few adjustments had to be made to the capturing system in order to adequately describe the CT finding. Whilst the statistical significance of the study proves to be limited by low numbers, the CT chest findings and patient demographics appear to be in keeping with the global disease profile produced in other studies. The endemic disease profile in our cohort, namely high HIV and PTB prevalence, fails to produce a significantly unique radiographic profile.

### What is known about this topic

- *More than half of patients who have survived COVID-19 infection-related admission have CT abnormalities at three months follow up;*
- *The most common findings are mixed reticular and ground glass parenchymal changes; some of which may or may not progress to fibrosis*.

### What this study adds

- *Our cohort is sampled from a South African population group with high background endemic TB and HIV prevalence with unique healthcare resource constraints, I.e. limited access to advanced imaging modalities and HCU/ICU care;*
- *We focus on CT chest findings in the immediate/early post-acute COVID-19 phase; as early as day 1 of the post-acute COVID-19 phase;*
- *Many global studies are centered on outpatient follow-up CT scans and typically range from 3-6 months since diagnosis*.
